# The fall of vulnerability to sleep disturbances in evening chronotypes when working from home and its implications for depression

**DOI:** 10.1038/s41598-022-16256-6

**Published:** 2022-07-18

**Authors:** Federico Salfi, Aurora D’Atri, Giulia Amicucci, Lorenzo Viselli, Maurizio Gorgoni, Serena Scarpelli, Valentina Alfonsi, Michele Ferrara

**Affiliations:** 1 Department of Biotechnological and Applied Clinical Sciences, University of L′Aquila, L′Aquila, Italy; 2 Department of Psychology, Sapienza University of Rome, Rome, Italy

**Keywords:** Human behaviour, Psychology and behaviour, Occupational health

## Abstract

Eveningness is distinctively associated with sleep disturbances and depression symptoms due to the misalignment between biological and social clocks. The widespread imposition of remote working due to the COVID-19 pandemic allowed a more flexible sleep schedule. This scenario could promote sleep and mental health in evening-type subjects. We investigated the effect of working from home on sleep quality/quantity and insomnia symptoms within the morningness-eveningness continuum, and its indirect repercussions on depressive symptomatology. A total of 610 Italian office workers (mean age ± standard deviation, 35.47 ± 10.17 years) and 265 remote workers (40.31 ± 10.69 years) participated in a web-based survey during the second contagion wave of COVID-19 (28 November–11 December 2020). We evaluated chronotype, sleep quality/duration, insomnia, and depression symptoms through validated questionnaires. Three moderated mediation models were performed on cross-sectional data, testing the mediation effect of sleep variables on the association between morningness-eveningness continuum and depression symptoms, with working modality (office vs. remote working) as moderator of the relationship between chronotype and sleep variables. Remote working was associated with delayed bedtime and get-up time. Working modality moderated the chronotype effect on sleep variables, as eveningness was related to worse sleep disturbances and shorter sleep duration among the office workers only. Working modality also moderated the mediation of sleep variables between chronotype and depression. The above mediation vanished among remote workers. The present study suggests that evening-type people did not show their characteristic vulnerability to sleep problems when working from home. This result could imply a reduction of the proposed sleep-driven predisposition to depression of late chronotypes. A working environment complying with individual circadian preferences might ensure an adequate sleep quantity/quality for the evening-type population, promoting their mental health.

## Introduction

Since the first months of 2020, the COVID-19 outbreak has deeply impacted the everyday life of the world population. After a summer period of reduced contagion and death rates, Winter 2020 was marked by a new exacerbation of the pandemic emergency^1^. This scenario radically affected the labor market as millions of workers were subjected to exceptional measures worldwide. The most widespread way to cope with the pandemic crisis has been a rapid transition to the remote work modality. According to a recent Eurofound report^2^, there was an upsurge in teleworking across all European countries during the COVID-19 pandemic. Approximately 40% of the European workforce began to work from home full-time. Similarly, in the United States, 35% of the population shifted from commuting to working remotely^3^.

Notwithstanding the large-scale nature of the remote working implementation, the consequences on sleep health of this unprecedented situation have been scarcely addressed. Remote working removed the need to spend time commuting between home and work, and it could be associated in some cases with greater flexibility of working hours. This situation allowed a better organization of the daily activities, leading to delayed and extended sleep time^4–6^. Consistently, we recently reported a beneficial effect of working from home on sleep quality, insomnia symptoms, and sleep duration among a large sample of the Italian population during the first contagion wave of COVID-19^6^. A positive effect of the transition to remote working on sleep quality and duration was also documented by other investigations^4,5,7,8^. However, some reports suggested that sleep quality^9^ and insomnia^10^ could worsen while working from home. The inconsistencies could be attributed to the lack of an evaluation of possible circadian typology effects in the available studies, considering that chronotype has been demonstrated to modulate the influence of the working schedule on sleep quality and duration^11,12^.

In our modern society, the issue of the misalignment between the daily social/working schedule and the internal biological clock is a long-standing controversy^13,14^. In 2006, Wittmann and colleagues^13^ coined the term “*social jetlag*” to give a face to this phenomenon. Consistent evidence pointed to a reduction of the *social jetlag* among the general population during the pandemic, when weaker social and working obligations led to a loosening of rigorous sleep/wake schedules^4,5,8,15,16^. Remarkably, *social jetlag* is intrinsically linked with the circadian typology, being typically more pervasive in the evening chronotype (the so-called “*owls*”). Among this group of people, who tend to go to bed and wake up later in a free-living condition, the mismatch between the endogenous biological and the exogenous social clock is the most pronounced^14^. This scenario lead to an accumulated sleep debt and more sleep problems during the working days in the evening chronotype^17–19^.

An adequate quantity/quality of sleep is crucial for emotional regulation^20,21^ and to preserve mental health^22,23^, and an extensive literature supports a determining role of both sleep disturbances and short sleep duration in the onset and exacerbation of depressive symptoms^24–28^. In this view, it is unsurprising that the evening chronotype has been systematically associated with a mood disturbance propensity^17,29^. Indeed, several recent reports suggested a causal role of sleep problems in accounting for the association between eveningness and depression^30–36^. On the other hand, people tending to go to bed and wake up earlier (“*larks*”) are less affected by *social jetlag*, having their sleep–wake rhythms aligned with the common social clock. This situation results in less severe sleep problems and depression symptoms among morning-type people^17^.

The large-scale transition toward remote working during the pandemic represented an unprecedented open-air laboratory to study the relationship between chronobiology and sleep health in a naturalistic environment. The current period emerges as an ideal context to address whether a more flexible working routine could influence sleep quality/quantity of the different circadian typologies and modify the mediating role of sleep between chronotype and depression.

In the present cross-sectional study, we investigated the effect of working from home during the second wave of the COVID-19 outbreak (28 November–11 December 2020) on sleep health/habits of almost nine hundred Italian workers placed along the morningness-eveningness continuum. We evaluated the moderator effect of the working modality (office vs. remote working) on the relationship between chronotype and sleep quality, insomnia symptoms, and sleep duration. We expected to confirm the well-known propensity of the evening-type people to experience sleep problems in the office working group. Meanwhile, we hypothesized that working from home could be associated with specific sleep benefits among the *owls*, flattening the difference in sleep disturbances among the different circadian typologies.

Finally, considering the causal role of sleep disturbances and duration in depressive symptomatology, we investigated the mediation role of sleep in the relationship between chronotype and depression symptoms, evaluating potential differences between office and remote workers. We expected to confirm a significant role of sleep disturbances/duration in accounting for the higher vulnerability to depression of the evening-typology among the office workers. On the other hand, we hypothesized that the mediation effect of sleep could be weakened in the group who worked from home.

## Methods

### Participants and procedure

The present study belongs to a research project aimed at understanding the consequences of the COVID-19 pandemic on sleep and mental health of the Italian population^6,37^. A total of 8,798 subjects participated in a web-based survey during the lockdown period due to the first contagion wave of COVID-19 (25 March–7 April 2020)^6^. A second survey wave was carried out during the second contagion wave (28 November–11 December 2020) by inviting respondents via email. A total of 2013 Italian citizens participated in this follow-up assessment^37^. Cross-sectional data reported in the present study are referred to the workers (N = 875; mean age ± standard deviation, 36.93 ± 10.57 years; range, 20–76 yrs; 729 females) who participated in the second survey wave. The selected sample comprised 610 full-time office workers (35.47 ± 10.17 yrs; 20–68 years; 515 females) and 265 full-time remote workers (40.31 ± 10.69 yrs; 23–76 years; 214 females). In the present study, we evaluated chronotype, sleep quality, insomnia symptoms, and depressive symptomatology through standard validated questionnaires [Morningness-Eveningness Questionnaire-reduced version (MEQr^38^), Pittsburgh Sleep Quality Index (PSQI^39^), Insomnia Severity Index (ISI^40^), Beck Depression Inventory-second edition (BDI-II^41^)].

The Institutional Review Board of the University of L’Aquila approved the study (protocol n. 43,066/2020), which was carried out according to the principles established by the Declaration of Helsinki. Online informed consent was obtained from all participants.

### Questionnaires

The MEQr is a 5-item questionnaire to assess the chronotype within the morningness-eveningness continuum. A higher total score (range, 4–25) is interpreted as a tendency to morningness and vice versa for eveningness. Cut-off scores are available to classify the chronotype groups (evening-type: 4–10; neither-type: 11–18; morning-type: 19–25). The PSQI is a widely used questionnaire to measure sleep quality. It comprised 19 items, and a higher total score indicates poorer sleep quality (range, 0–21). From the PSQI, we further extracted the answers to the items “sleep duration” (min), “bedtime” (hh:mm), and “get-up time” (hh:mm), which were used in the analyses described in the next paragraph and in the “Supplementary Information” section. The ISI is a 7-item questionnaire to evaluate the severity of insomnia symptoms, where higher scores point to more severe insomnia symptoms (range, 0–28). The BDI-II is a screening instrument to assess the severity of clinical depression. A higher score ranging between 0 and 64 demonstrates more severe depression symptoms. The satisfactory psychometric proprieties of the Italian version of all the adopted questionnaires have been documented by previous studies confirming them as valid and reliable instruments to evaluate chronotype (MEQr^38^), sleep quality (PSQI^39^), insomnia symptoms (ISI^40^), and depression (BDI-II^42^).

### Statistical analysis

We computed group descriptive statistics (office working, remote working) for all the considered variables in the current study. We evaluated potential differences in gender composition between the two groups through *Chi*-square test. Moreover, we compared office and remote working groups on age and questionnaire scores (MEQr, PSQI, ISI, BDI-II), sleep duration (min), bedtime (hh:mm), and get-up time (hh:mm), using Mann–Whitney *U* test, considering the violation of normality/heteroscedasticity assumptions. All tests were two-tailed, and a *p*-value < 0.05 was considered significant. Harman's one-factor test did not show any common method bias in our data.

According to the research hypotheses, three moderated mediation analyses were run using model 7 of PROCESS macro (version 3.5^43,44^) for SPSS (version 22.0). Model 7 assumes that the first stage of the mediation model is moderated. We included MEQr score as independent variable, each sleep outcome [PSQI and ISI scores, sleep duration (min)] as individual mediator, and BDI-II score as dependent variable. All the above outcomes were analyzed as continuous variables. The direction of the effects was established by relying on a consistent meta-analytic literature on longitudinal epidemiological studies supporting a causal role of sleep problems in the development of depressive symptoms^24,26,27^, as well as on several studies proposing a mediation effect of sleep variables between chronotype and depression^30–36^. We separately included sleep variables as mediators in each model due to violations of the assumptions for alternative parallel mediation analysis^44^. Firstly, mediators are intrinsically related to each other as they evaluate overlapping constructs. Moreover, mediators are strongly correlated (PSQI scores and ISI scores: *r* = 0.80, *p* < 0.001; PSQI scores and sleep duration: *r* = − 0.67, *p* < 0.001; ISI scores and sleep duration: *r* = − 0.53, *p* < 0.001). This evidence constitutes a second violation of the mediation assumptions because their parallel inclusion in a model would give rise to a multicollinearity problem, which affects the estimation of their partial relationships with the outcome variable^44^.

**Figure 1:**
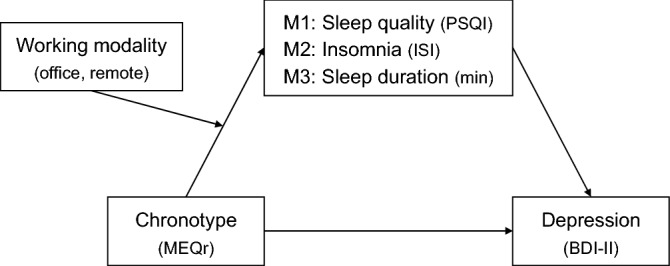
The three theoretical moderated mediation models tested (M1, M2, M3). Three mediators (sleep quality, insomnia symptoms, sleep duration) are hypothesized to mediate the relationship between morningness-eveningness continuum and severity of depression symptoms in a context where working modality (office working, remote working) moderate the effect of chronotype on sleep variables. Each model was adjusted for age and gender. *Abbreviations:* PSQI, Pittsburgh Sleep Quality Index; ISI, Insomnia Severity Index; MEQr, Morningness-Eveningness Questionnaire-reduced version; BDI-II, Beck Depression Inventory-second edition.

The working modality factor (office working, remote working) was assumed as a moderator of the first path of the mediation models (chronotype → sleep variables), and it was entered as a dichotomous dummy variable (office working: 0, remote working: 1). Finally, since previous studies indicated that sleep problems and depressive symptoms correlate with age and gender^45–52^, we included them in the models as continuous and dummy coded (female: 0, male: 1) covariates, respectively. A summary of the three theoretical models tested is provided in Fig. 1. Simple slope analyses were performed to explore the nature of the significant interactions between working modality (office working, remote working) and chronotype in predicting sleep variables (sleep quality, insomnia symptoms, sleep duration). The statistical significance of the conditional indirect effects was ascertained by means of 5,000 bootstrap samples to create bias-corrected confidence intervals (*CIs*: 95%) with heteroscedasticity-consistent standard errors (SEs). Moderated mediation models were considered significant and accepted when the interval between the 95% bootstrapped lower limit (*BootLLCI*) and upper limit of CIs (*BootULCI*) of the index of moderated mediation (the difference between conditional indirect effects) does not contain 0.

Finally, to further clarify how remote working affected the sleep schedule within the morningness-eveningness continuum, we analyzed how chronotype scores interact with working modality in predicting sleep onset and offset time. Therefore, two explorative moderation models were tested, assuming working modality as moderator of the effect of chronotype on bedtime and get-up time (see Supplementary Fig. S1 online).

## Results

### Characteristics of participants

The demographic composition of the two groups (office working, remote working) and descriptive statistics of the main study variables are shown in Table 1.

**Table 1:** Characteristics of participants divided by working modality (office, remote).

	Working modality		*Statistic*	*df*	*p*
	Office [N = 610 (69.7%)]	Remote [N = 265 (30.3%)]			
	*Mean* ± *SD*	*Mean* ± *SD*			
**Gender**					
Male	95 (15.6%)	51 (19.2%)	1.791*	1	0.181
Female	515 (84.4%)	214 (80.8%)			
Age	35.467 ± 10.174	40.309 ± 10.694	58,326^†^	873	**< 0.001**
MEQr score	15.867 ± 3.494	15.430 ± 3.850	76,014.5^†^	873	0.160
PSQI score	6.693 ± 3.504	7.015 ± 3.598	76,367.5^†^	873	0.192
ISI score	7.428 ± 5.268	7.815 ± 5.356	77,398^†^	873	0.318
Sleep duration (min)	403.365 ± 66.462	401.624 ± 64.434	79,169^†^	873	0.626
Bedtime (hh:mm)	23:11 ± 1:05	23:37 ± 1:16	63,901^†^	873	**< 0.001**
Get-up time (hh:mm)	7:13 ± 1:03	7:41 ± 1:09	56,625^†^	873	**< 0.001**
BDI-II score	11.141 ± 8.620	11.660 ± 9.698	79,897^†^	873	0.787

Results of the comparisons between the working modality groups are also shown.

*Chi-square, ^†^Mann–Whitney U.

*SD* standard deviation, *df* degrees of freedom, *PSQI* Pittsburgh sleep quality index, *ISI* insomnia severity index, *MEQr* morningness-eveningness questionnaire-reduced version, *BDI-II* beck depression inventory-second edition.

Significant values are in bold.

The two samples did not differ in MEQr, PSQI, ISI, and BDI-II scores, as well as in sleep duration and gender proportion. However, the office working group was significantly younger and reported earlier bedtime and get-up time.

### Moderated mediation analyses

The regressions on mediators (PSQI score, ISI score, sleep duration) including age, gender (male, female), MEQr scores, working modality (office working, remote working), and the interaction between MEQr scores and working modality as predictors were significant for each model (Model 1: *R*^2^ = 0.069, *F* = 12.856, *p* < 0.001; Model 2: *R*^2^ = 0.063, *F* = 11.617, *p* < 0.001; Model 3: *R*^2^ = 0.094, *F* = 18.057, *p* < 0.001). Likewise, the regressions on BDI-II scores including age, gender (male, female), MEQr scores, and sleep variables (Model 1: PSQI score; Model 2: ISI score; Model 3: sleep duration) as predictors were significant for all the models (Model 1: *R*^2^ = 0.289, *F* = 88.123, *p* < 0.001; Model 2: *R*^2^ = 0.373, *F* = 129.62, *p* < 0.001; Model 3: *R*^2^ = 0.135, *F* = 33.947, *p* < 0.001). As showed in Table 2, older age was associated with lower sleep quality, more severe insomnia, shorter sleep duration, and lower depressive symptoms in all the models. Male subjects reported better sleep quality and less severe insomnia symptoms than females, while no difference in sleep duration between genders emerged. Men showed a lower severity of depression in each model.

**Table 2:** Unstandardized effects (*B*), *t*-value, and significance of the covariates (age, gender) for the three models, including sleep quality (PSQI score; Model 1), insomnia symptoms (ISI score; Model 2), and sleep duration (min; Model 3) as mediators.

Covariate effects	*B*	*t*	*p*
**Model 1**			
Age → PSQI	0.051	4.480	**< 0.001**
Gender* → PSQI	− 1.230	− 3.915	**< 0.001**
Age → BDI-II	− 2.188	− 3.120	**0.002**
Gender* → BDI-II	− 0.067	− 2.690	**0.007**
**Model 2**			
Age → ISI	0.052	3.040	**0.002**
Gender* → ISI	− 1.943	− 4.112	**< 0.001**
Age → BDI-II	− 0.054	− 2.336	**0.019**
Gender* → BDI-II	− 1.820	− 2.764	**0.006**
**Model 3**			
Age → Sleep duration	− 1.834	− 8.752	**< 0.001**
Gender* → Sleep duration	− 6.908	− 1.196	0.232
Age → BDI-II	− 0.071	− 2.493	**0.013**
Gender* → BDI-II	− 3.998	− 5.217	**< 0.001**

*Female was used as reference for “Gender” factor.

*PSQI* Pittsburgh sleep quality index, *ISI* insomnia severity index, *BDI-II* beck depression inventory-second edition.

Significant values are in bold.

As reported in Table 3, both the MEQr scores and the sleep variables (PSQI score, ISI score, sleep duration) were significantly associated with the BDI-II scores in all the models (direct effects). Tendency to eveningness, lower sleep quality, more severe insomnia, and shorter sleep duration predicted greater depressive symptoms. The conditional direct effects at the value of the moderator (office working, remote working) indicated that the tendency to morningness was associated with better sleep quality, lower severity of insomnia symptoms, and longer sleep duration in the office working group. On the other hand, no significant relationship between chronotype and sleep variables emerged among the remote workers.

**Table 3:** Direct effects and conditional direct effects at the value of the moderator (office working, remote working) for the three models, including sleep quality (PSQI score; Model 1), insomnia symptoms (ISI score; Model 2), and sleep duration (min; Model 3) as mediators, whilst accounting for the effects of age and gender.

Direct effects	*B*	*t*	*p*
**Model 1**			
MEQr → BDI-II	− 0.274	− 3.724	**< 0.001**
PSQI → BDI-II	1.245	16.613	**< 0.001**
**Model 2**			
MEQr → BDI-II	− 0.233	− 3.376	**< 0.001**
ISI → BDI-II	0.971	20.775	**< 0.001**
**Model 3**			
MEQr → BDI-II	− 0.445	− 5.560	**< 0.001**
Sleep duration → BDI-II	− 0.038	− 8.535	**< 0.001**

Conditional direct effects	*B*	*t*	*p*
**Model 1**			
Office working: MEQr → PSQI	− 0.247	− 6.151	**< 0.001**
Remote working: MEQr → PSQI	− 0.101	− 1.825	0.068
**Model 2**			
Office working: MEQr → ISI	− 0.374	− 6.200	**< 0.001**
Remote working: MEQr → ISI	− 0.141	− 1.696	0.090
**Model 3**			
Office working: MEQr → Seep duration	3.023	4.102	**< 0.001**
Remote working: MEQr → Sleep duration	0.151	0.149	0.881

*PSQI* Pittsburgh sleep quality index, *ISI* insomnia severity index, *MEQr* morningness-eveningness questionnaire-reduced version, *BDI-II* beck depression inventory-second edition.

Significant values are in bold.

**Figure 2:**
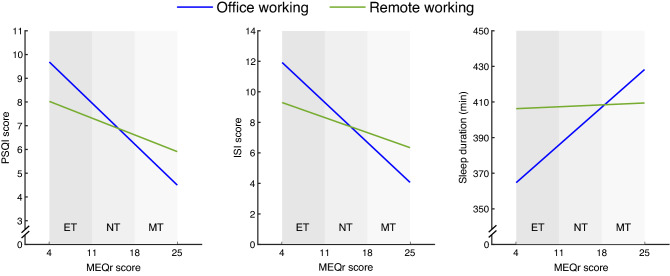
Simple slope analyses of the interaction between MEQr scores and working modality [office working (blue line), remote working (green line)] on sleep quality (PSQI score), insomnia symptoms (ISI score), and sleep duration (min). Gray bands discriminate chronotypes according to the validated cut-off scores. *Abbreviations:* ET, evening type; NT, neither type; MT, morning type; PSQI, Pittsburgh Sleep Quality Index; ISI, Insomnia Severity Index; MEQr, Morningness-Eveningness Questionnaire-reduced version.

The working modality moderator was significant in each model (Table 4), indicating that remote workers reported higher sleep quality, lower insomnia symptoms, and longer sleep duration than the office working group. The interaction between MEQr scores and the working modality moderator was significant in each model, suggesting a different linear relationship between chronotype and sleep variables comparing the office and remote working groups (Fig. 2).

**Table 4:** Moderator and interaction effects for the three models, including sleep quality (PSQI score; Model 1), insomnia symptoms (ISI score; Model 2), and sleep duration (min; Model 3) as mediators, whilst accounting for the effects of age and gender.

Moderator effects	*B*	*t*	*p*
**Model 1**			
Working modality* → PSQI	− 2.242	− 2.062	**0.039**
**Model 2**			
Working modality* → ISI	− 3.555	− 2.175	**0.030**
**Model 3**			
Working modality* → Sleep duration	53.026	2.654	**0.008**

Interaction effects	*B*	*t*	*p*
**Model 1**			
Working modality* X MEQr → PSQI	0.146	2.158	**0.031**
**Model 2**			
Working modality* X MEQr → ISI	0.233	2.291	**0.021**
**Model 3**			
Working modality* X MEQr → Sleep duration	− 2.872	− 2.309	**0.012**

*Office working was used as reference for “Working modality” factor.

*PSQI* Pittsburgh sleep quality index, *ISI* insomnia severity index, *MEQr* morningness-eveningness questionnaire-reduced version.

Significant values are in bold.

**Figure 3:**
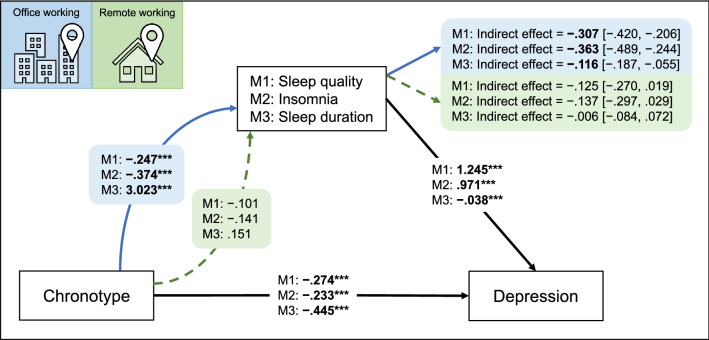
Summary of the results of the three moderated mediation models (M1, M2, M3). The figure reports the unstandardized coefficients of direct effects, conditional direct effects at the value of moderator, and conditional indirect effects with bootstrapped computed confidence intervals for the two levels of moderator [office working (blue arrow/area), remote working (green arrow/area)]. Significant effects are reported in bold, and the significance level of direct effects is indicated with asterisks (***p < .001).

Finally, as reported in Fig. 3, all the conditional indirect effects were significant for the office working group, indicating that the sleep variables partially mediated the effect of chronotype on depression symptoms. On the other hand, no significant indirect effect was detected in the remote working group. Consistently, the index of moderated mediation was significant in each model (Model 1: 0.182 [0.008, 0.361]; Model 2: 0.227 [0.031, 0.430]; Model 3: 0.110 [0.015, 0.221]), indicating that working from home suppressed the mediation effect of sleep variables on the association between chronotype and depression. Control analyses including occupation and educational level in the moderated mediation models confirmed all the reported results (data not shown).

## Discussion

The COVID-19 emergency pervasively impacted the daily routine of millions of workers worldwide. Consistent with European and American reports during the pandemic^2,3^, three out of ten individuals in our sample worked full-time from home. In line with recent investigations^4–6,53^, the remote working group showed a general delay in bedtime and get-up time. We hypothesized that evening-type subjects could have benefited from such a scenario, as their sleep time was better aligned with the endogenous circadian phase than the office working sample. The analyses supported our prediction: the tested models revealed a significant interaction between chronotype scores and working modality (office, remote) in predicting sleep variables. Remarkably, we showed that the well-documented relationship between chronotype and sleep problems/duration^17–19^ was limited to the office working group. Therefore, our findings suggest that the propensity to sleep problems and shorter sleep duration of evening-type people may not depend on chronotype per se*.* Eveningness could represent a risk factor only in the office working condition.

The outcomes of the additional models (see Supplementary Fig. S2 online) contributed to further clarifying the pattern of results. We showed that working from home influenced the relationship between chronotype and get-up time but did not affect the association with bedtime. Specifically, eveningness was related to a stronger tendency to get-up later in the remote working group than in the office working sample. On the other hand, the inclination of evening-type people to go to bed later than morning-type was comparable in the two working modality groups. Therefore, later bedtimes were not adequately compensated by later get-up times when participants had to reach the workplace, giving rise to the shorter sleep duration tendency shown by the late chronotypes. On the other hand, strengthening the association between get-up times and chronotype scores implies that the *owls* slept more when they worked from home, leading to the extinction of the sleep duration differences between circadian typologies.

Our findings are consistent with studies showing that chronotype modulates the effect of the working schedule on sleep patterns^11,12^. Late chronotypes are characterized by shorter sleep duration and more severe sleep disturbances compared with early ones when working in the morning^11^. Consistently, a chronotype-based working routine was associated with increased sleep duration and quality by reducing the *social jetlag* among the evening-type population^12^.

Several studies demonstrated a positive effect of working from home on sleep patterns during the current pandemic^4,5,7,8,53^. This literature is consistent with investigations on the student population under remote learning due to the COVID-19 emergency, where participants delayed their sleep time and reported longer sleep duration and improved sleep quality^54–57^. Notwithstanding the lack of an evaluation of possible differential effects as a function of circadian typology in these studies, the results were interpreted as a general tendency to synchronize the sleep/wake cycle with the individual biological clock when daily schedules are less strongly dictated by the office/school hours. Interestingly, we did not find significant differences in mean sleep quality, sleep duration, and severity of insomnia symptoms in the preliminary direct between-group comparisons. However, the beneficial effect of remote working on sleep emerged by including the interaction between chronotype scores and working modality in the models. This evidence could account for some of the inconsistencies in the literature addressing remote working effects during the pandemic period^9,10^. The individual circadian preference could act as a confounding variable, resulting in misleading conclusions when studying the consequences of the working modality on sleep health. Therefore, we caution that future studies in this field duly consider chronotype and its interaction with both working modality and schedule.

As far as the depressive symptomatology is concerned, we confirmed the tendency of the evening-types to experience more severe symptoms^17,29^, as well as the well-documented relationship between both sleep problems and short sleep duration and more severe depression symptoms^24–28^. Meanwhile, the loosening of the proposed association between sleep disturbances/duration and chronotype in the group who worked from home corroborated the second goal of this study: determining whether remote working affected the mediation role of sleep between circadian typologies and depression symptoms.

The three moderated mediation models demonstrated that poorer sleep quality, more severe insomnia symptoms, and shorter sleep duration could partially explain the tendency of the late chronotypes to experience depression, but only when they had to reach the workplace.

This outcome is consistent with a growing literature supporting a causative role of sleep disturbances and shorter sleep duration in explaining the eveningness susceptibility to depressive symptomatology^30–35^. On the other hand, we showed that the sleep-dependent vulnerability to depression of late chronotypes disappeared under remote working. Therefore, the improvement of sleep problems while working from home could indirectly promote the mental health of evening-type participants, influencing their predisposition to depressive symptoms.

The present results were obtained in a sample where older respondents and females experienced poorer sleep quality and more severe insomnia symptoms, women reported higher depressive symptomatology, and younger people slept longer and showed more severe depression. These results are consistent with an extensive pre-pandemic and pandemic literature showing a tendency of women to report worse sleep disturbances^6,45,47,48,52^ and depression symptoms^46,47,52^, as well as the predisposition of older age to experience poorer sleep quality^6,48,58,59^, more insomnia^6,50^, shorter sleep duration^51,58^, and a lower predisposition to mood disorders^51,52^.

The results of this study solicit a discussion at the community level. Our modern society forces many employees to fit a “standard” work schedule typically oriented to morningness. Social pressure imposes to get up early in the morning beginning from the school period; this situation limits the time available for sleep and leads adolescents to be awake at an inappropriate circadian phase^60^. This issue spans to adulthood as early morning working is associated with inadequate sleep, more sleep problems, and fatigue among the general population^61,62^. This situation is even more pronounced when the large and intrinsic variability in the biological circadian predispositions is considered^11,12^, configuring a latent penalization of evening-type people. Considering the individual circadian predisposition in managing the working environment could promote late chronotypes’ sleep and mental health.

The vaccination campaign and the gradual mitigation of the pandemic crisis are leading people to resume their pre-pandemic working routine worldwide. In this vein, our results could have large-scale implications spanning the post-pandemic period, considering that circadian predisposition has a substantial genetic component^63,64^ that could be hardly manipulated, and the current literature estimated a 10–20% prevalence of *owls* among the adult population^6,65–69^, which is also higher among young people^17,70^. The outcomes of the present study should be taken into account when designing remote working policies during the current pandemic, as well as in the post-covid era.

## Limitations

Our pattern of results was obtained in a large sample of workers. Moreover, the inclusion of demographic factors (age and gender) in all the tested models confuted the possibility that the younger age of the office working group could have biased our results. However, some limitations should be acknowledged. We adopted a cross-sectional design, the sample comprised a higher prevalence of women, and we did not collect information about pre-pandemic experience of remote work. Furthermore, our findings relied on regression analyses so that the direction of the effects could be only hypothesized. Consequently, caution is required in interpreting the indirect effects, considering the potential bidirectionality between sleep disturbances and depressive symptoms^71^. A longitudinal analysis might confirm our results clarifying the causal relationship between the investigated variables and evaluating the effects of a change of working modality in a prospective within-subject study design. Additionally, we assessed chronotype, sleep variables, and depression using self-report retrospective questionnaires. Future research should adopt ecological momentary assessment technologies to minimize recall bias and maximize ecological validity to provide more reliable results^72^. Moreover, our evaluation of sleep habits did not discriminate between workdays and free days. However, a sleep evaluation targeted on workdays could have provided even stronger evidence of the interaction between chronotype and working modality, although PSQI scores predominantly reflect sleep quality/patterns of workdays^73^. Finally, an ad hoc evaluation of the *social jetlag* phenomenon through, e.g., the Munich Chronotype Questionnaire^74^, might have contributed to better understand the effect of working from home during this unprecedented pandemic period.

## Supplementary Information

Supplementary Information.

## Data Availability

The datasets analyzed during the current study are available from the corresponding author on reasonable request.
