# Supplementary material for: The fall of vulnerability to sleep disturbances in evening chronotypes when working from home and its implications for depression
Source: Sci Rep. 2022 Jul 18;12:12249. doi: 10.1038/s41598-022-16256-6 (PMC9293935; doi:10.1038/s41598-022-16256-6)
Supplement: Supplementary file 1 — Supplementary Information. [file 41598_2022_16256_MOESM1_ESM.pdf]

The fall of vulnerability to sleep disturbances in evening chronotypes when working from home and its implications for depression

Federico Salfi[1], Aurora D'Atri[1], Giulia Amicucci[1,2], Lorenzo Viselli[1], Maurizio Gorgoni[2], Serena Scarpelli[2], Valentina Alfonsi[2], and Michele Ferrara[1]*

[1]Department of Biotechnological and Applied Clinical Sciences, University of L'Aquila, L'Aquila, Italy

[2]Department of Psychology, Sapienza University of Rome, Rome, Italy

*Corresponding author

Prof. Michele Ferrara, Ph.D.

Department of Biotechnological and Applied Clinical Sciences

University of L'Aquila

Via Vetoio (Coppito 2)

67100 Coppito (AQ)

Italy

michele.ferrara@univaq.it

Supplementary Information

Two additional moderation models were tested using model 1 of PROCESS macro (version 3.5[1,2]) for SPSS (version 22.0). We assumed that working modality (office working, remote working) moderated the effect of chronotype on bedtime and get-up time (Fig. S1). Both the models included the covariance of age and gender.

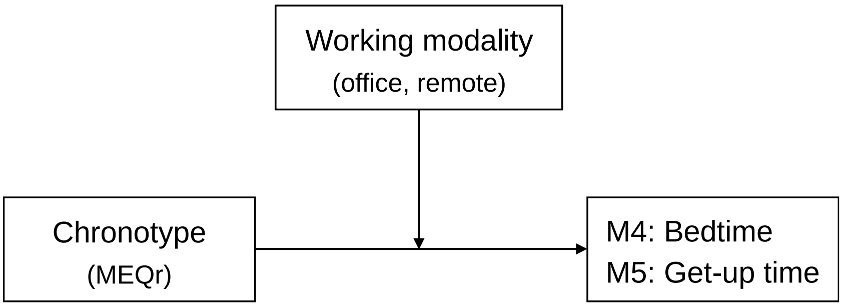

Figure S1. The two additional moderation models tested (M4, M5). Chronotype is hypothesized to predict bedtime and get-up time, while the working modality (office working, remote working) moderated this relationship.
Abbreviations: MEQr, Morningness-Eveningness Questionnaire-reduced version.

The regressions on bedtime and get-up time were significant (Model 4: R^2 = .247, F = 56.852, p < .001; Model 5: R^2 = .274, F = 65.685, p < .001). The covariate effect of "gender" emerged significant for the bedtime variable (B = 14.51 min, t = 2.630, p = .009), indicating that female respondents went to bed earlier. Age was a significant predictor of get-up time (B = −1.58 min, t = −8.509, p < .001), indicating that older age was associated with earlier get-up time.

The conditional direct effects at both values of the moderator (office working, remote working) were significant in both the models. In particular, MEQr score predicted significantly bedtime (office working: B = −9.80 min, t = −10.029, p < .001; remote working: B = −7.92 min, t = −11.154, p < .001) and get-up time (office working: B = −8.50 min, t = −9.317, p < .001; remote working: B = −6.05 min, t = −9.221, p < .001), confirming the tendency to delayed sleep time of evening-type people in both the working modality conditions. The "working modality" moderator was significant in both the models (M4: B = 51.55 min, t = 2.682, p = .008; M5: B = 69.27 min, t = 3.874, p = .001), showing that people working from home went to bed and got-up later. Finally, the significant interaction between "working modality" and MEQr score was limited to

the get-up time variable (M4: $B = -1.87$ min, $t = -1.564$, $p = .118$; M5: $B = -2.30$ min, $t = -2.130$, $p = .033$), highlighting that the relationship between chronotype and rising time was stronger in those who worked from home than in the office working condition (Fig. S2).

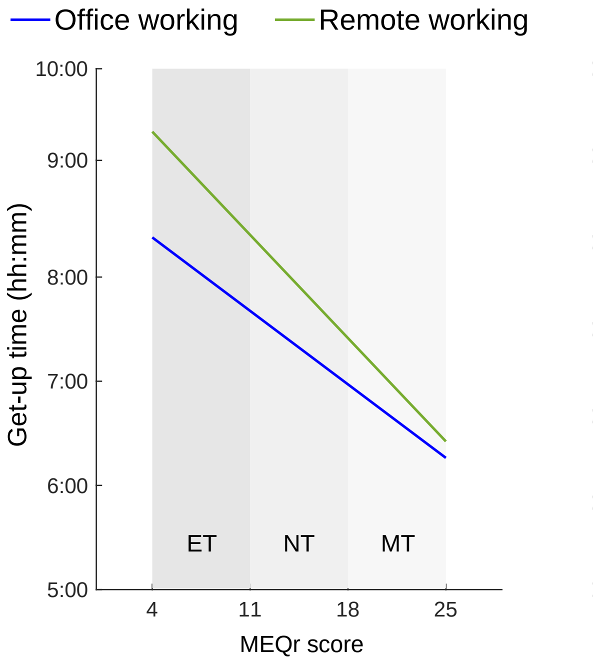

Figure S2. Simple slope analysis of the interaction between MEQr scores and working modality [office working (blue line), remote working (green line)] on get-up time (hh:mm). Gray bands identify chronotypes according to the validated cut-off scores.
Abbreviations: ET, evening type; NT, neither type; MT, morning type; MEQr, Morningness-eveningness questionnaire-reduced version.

References

1. Hayes, A. F. Introduction to mediation, moderation, and conditional process analysis: a regression-based approach in *Methodology in the Social Sciences, 2nd edn.* (Guilford Publications Inc, 2017).

2. Hayes, A. F. *Introduction to mediation, moderation, and conditional process analysis A regression-based approach*. (The Guilford Press, 2013).
